# What can we expect from medical graduates? Empirical survey on the performance of Core EPAs in the first days of residency

**DOI:** 10.1186/s12909-020-02376-y

**Published:** 2020-11-23

**Authors:** Ylva Holzhausen, Asja Maaz, Yadira Roa-Romero, Harm Peters

**Affiliations:** 1 Dieter Scheffner Centre for Medical Education and Educational Research, Dean’s Office of Study Affairs, Charité - Universitätsmedizin Berlin, Berlin, Germany; 2 Department of Quality Management (QM) Teaching and Learning, Dean’s Office of Study Affairs, Charité - Universitätsmedizin Berlin, Berlin, Germany

**Keywords:** Entrustable professional activities, Undergraduate education, Medical graduates, Residents

## Abstract

**Background:**

Core Entrustable Professional Activities (EPAs) have been defined to specify the performance expectations for entering residents worldwide. The content of these EPAs was elaborated and validated primarily via medical expert consent approaches. The present study aims to collect empirical information on the actual task performance and supervision level of entering residents as a complementary methodological approach to enhance the content validity of a set of institutional EPAs.

**Methods:**

In the summers of 2017 and 2018, Charité medical graduates (*n* = 720) received a post-graduation survey by mail. The questionnaire covered the performance of Core EPAs, Core procedures and more advanced EPAs. Graduates were asked how frequently they had performed the respective EPAs since the start of residency and under what level of supervision. We expected the large majority of graduates (> 75%) to have performed the Core EPAs and procedures under at least indirect supervision.

**Results:**

In total, 215 graduates (30%) returned the questionnaire, and 131 (18%) surveys could be included in the data analysis. The majority of participants were female (63%) and worked in hospitals (50%) or in university medical centres (30%) across various medical disciplines. Among the Core EPAs, 10 out of 11 tasks had been performed by more than 75% of graduates since the start of residency, 9 under indirect supervision. Regarding the Core procedures, only 3 out of 13 procedures had been performed by the large majority of graduates under indirect supervision, and 10 procedures had not been carried out by at least one-third of participants. Among the 5 advanced EPAs, none of 5 had been performed by more than 75% of the participants since the start of residency, and 4 had been carried out by 50% under indirect supervision.

**Conclusions:**

The results of this study largely and complementarily confirm the validity of the defined Core EPAs representing realistic expectations for entry into residence at our institution. The low actual performance rate of Core procedures serves to stimulate an institutional discussion on their adjustment to better match the workplace reality.

**Supplementary Information:**

The online version contains supplementary material available at 10.1186/s12909-020-02376-y.

## Background

Entry into residency is characterized by a sudden and often overwhelming increase in responsibilities for graduating physicians with respect to the care of patients. To ease this critical transition, medical education experts have developed Core Entrustable Professional Activities (EPAs) for entry into residency [1–5]. The content elaboration and validation of such Core EPAs did not involve explicit empirical data or research on the tasks entering residents actually perform. However, such real-world data from clinical workplaces could be used for triangulation of the performance expectations with experts’ opinions by providing complementary evidence of their content validity or, in the case of divergence, an opportunity for the re-adjustment of the Core EPAs.

The EPA concept builds on authentic tasks that trainees perform in the workplace under varying levels of supervision [6, 7]. Depending on the level of competence of the trainees, professional activities can be gradually entrusted to them. The idea behind the EPA concept is to define those tasks trainees are commonly expected to perform at the end of a training phase without supervision and to implement these as outcomes for the curriculum.

The beginning of residency is educationally very dynamic, as the responsibilities of entering residents increase markedly in a short period, from smaller units of work, such as history taking and the physical examination of new patients, to conducting and managing the complete admission of a patient or even conducting a night shift with complete responsibility for the care of patients. Core EPAs for entry into residency aim to define the performance expectations for beginning residents, irrespective of the discipline chosen, and are implemented as outcomes in undergraduate medical curricula. The content of the developed Core EPA frameworks was generally defined by various expert groups, i.e., medical educators or experienced physicians, using iterative group discussions and various consent-finding methods [1–5]. Overall, the Core EPAs have much in common; however, they also involve relevant differences in content. These differences are related to the kind and number of professional tasks, their specifications and limitations, and the respective levels of supervision [5]. These differences may reflect variations in workplace practices in different contexts or disciplines but may also be indicative of a need to further adjust the content. Notably, the existing Core EPAs were generally defined by majority voting procedures. Thus, there were possibly several experts in the groups who did not agree that the defined EPAs reflect the expectations for entering residency. In addition, others have argued that the breadth and depth of the Core EPAs are too narrow to reflect the expectations and workflows in clinical settings, calling for more aspirational and less granular EPAs [8, 9].

The Core EPA concept implies that the professional activities included, and the supervision levels assigned reflect the requirements and practices in the real-life work context for entering residents. Gaining insights into and empirical information on the workplace reality of entering residents could provide additional validating evidence for the Core EPAs and could be used to adjust them. To date, there is only sparse, insufficiently detailed information and research available on what entering residents actually do in the first weeks and months of residency. Existing studies on the experiences of beginning residents have assessed the needed supervision level or residents’ preparedness to perform the Core EPAs without direct supervision [10, 11]. The results show that the majority of residents show high confidence, identifying only a few EPAs that the majority do not feel safe to perform without direct supervision. Other studies assessed residents’ general confidence or preparedness to perform the AAMC Core EPAs, without specifying the level of needed supervision [12, 13].

The aim of this study was to explore the actual workplace involvement of entering residents on the basis of a set of Core EPAs for entering into residency recently defined at our institution, the Charité – Universitätsmedizin Berlin (Charité). A section of questions regarding the professional tasks performed, how frequently and under what level of supervision was incorporated into the regular post-graduation survey. We further explored the degree to which the EPA variables are interrelated.

## Method

### Previous work

At the Charité, institutional Core EPAs were defined from 2014 to 2015 in a modified, three-round Delphi procedure with an expert group consisting of 45 experienced clinicians from various disciplines. Panel members were asked to rate the relevance of professional tasks for entering residents at the indirect supervision level as measures for Core EPA identification and validation. Consent was achieved when a content validity index (CVI) of ≥80% was reached. The process resulted in a set of Core EPAs and Core medical procedures [5, 14].

### Participants

In the summers of 2017 and 2018, Charité medical graduates (*n* = 720) received a regular post-graduation evaluation questionnaire by mail within 9 months after graduation. They were asked to answer the questionnaire and send it back to the research team at the Charité. Contacting graduates via e-mail was not possible as their institutional e-mail addresses expired 3 months after graduation. The study protocol received approval from the Charité data protection office (No. 567/16) and ethics board (No. EA4/051/20).

### Questionnaire

The questionnaire was developed by an institutional working group with colleagues from the department of Quality Management (QM) Teaching and Learning, the Dieter Scheffner Centre for Medical Education and Educational Research, the Institute of Medical Sociology and Rehabilitation Science and with medical students from the Charité. It comprised a broad spectrum of questions covering person-related data, the evaluation of the medical curriculum and the respondents’ experiences as graduates. The development of the questionnaire was distributed among the working group members according to their field of expertise. The questions were partly derived from previous questionnaires and partly newly created.

One section entailed questions regarding the Charité EPAs expected to be performed in the first days of residency (Additional File 1). Table 1 provides an overview of the 11 Core EPAs and 13 Core medical procedures that were asked about in the questionnaire and the corresponding terms used in this manuscript. The questionnaire also included five broader, less granular EPAs representing more advanced expectations for medical graduates. The questions about the EPAs were developed by the authors to characterize the real-life workplace involvement of graduates.

**Table 1:** Overview of EPAs used in the questionnaire

	Terms used in the manuscript	EPA descriptions used in the questionnaire
**Core EPAs**	History, physical and synthesis	Take a medical history, perform a physical examination and summarize the results in a structured manner (typical presentation, common disease pattern)
	Diagnostic plan	Compile a diagnostic plan and initiate implementation (typical presentation, common disease pattern, typical course of disease; tiered diagnostics)
	Interpret test results	Interpret test results and initiate further steps (common diagnostic methods)
	Treatment plan	Compile a treatment plan and initiate implementation (common disease pattern, typical course of disease)
	Obtain Informed consent	Seek consent for medical procedures and diagnostics (inform patient about course, benefits, risks and alternatives)
	Inform and advise a patient	Inform and advise patients (common consolations, reasons and diseases)
	Present patient history	Present a patient history (structured; according to the target audience and situational requirements)
	Patient handover	Give or receive a patient handover (structured; according to the target audience and situational requirements)
	Patient report	Write and transmit a patient report (structured; transmit oneself or delegate)
	Act in emergency situations	Recognize an emergency situation and act upon it (estimate the degree of severity, provide on-the-spot aid, call for help)
	Evidence-based case presentation	Undertake an evidence-based patient case and initiate patient-specific implementation
**Core Procedures**	Venous blood sampling	Venous blood sampling
	Capillary blood sampling	Capillary blood sampling
	Peripheral catheter	Inserting a peripheral catheter
	Blood culture	Taking a blood culture
	Taking a smear	Taking a smear (oral, nasal, wound, anal, urogenital)
	Intracutaneous injection	Giving an intracutaneous injection
	Subcutaneous injection	Giving a subcutaneous injection
	Intramuscular injection	Giving an intramuscular injection
	Infusion	Giving an infusion
	Nasogastric tube	Placing a nasogastric tube
	ECG	Taking an ECG
	Bandage	Putting on or changing a bandage
	Prescription	Writing a prescription
**Advanced EPAs**	Complete patient admission	Manage an in-patient admission
	Ward round	Conduct a ward round in the hospital
	Complete patient discharge	Manage an in-patient discharge
	Weekend ward round	Conduct a weekend ward round in the hospital
	Late/night shift	Take a late/night shift (supervising physician available via telephone)

Participants were asked:

1) how many times they performed the activities (frequency of performing EPAs) and

2) to indicate the highest level of supervision under which they performed an EPA at least three times (experienced supervision level).

**Fig. 1:**
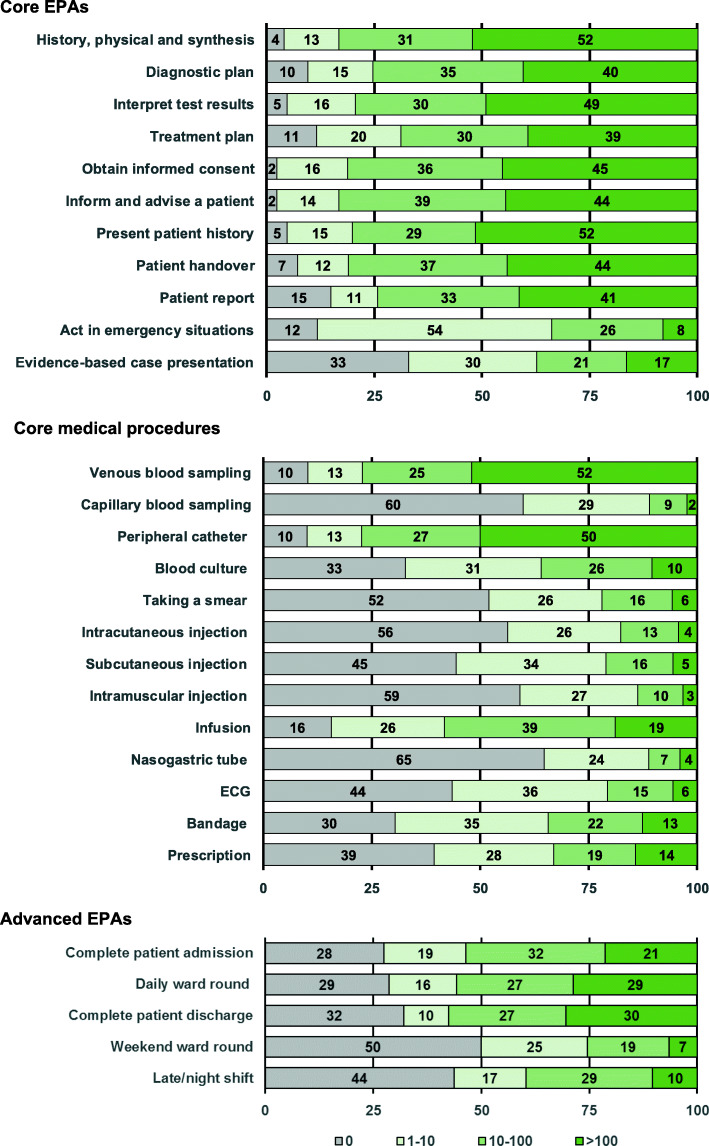
Frequency of performing EPAs. The figure depicts the valid percentage of participants performing the EPAs

The frequency was captured by the following response categories: 0; 1–5; 6–10; 11–25; 26–100; > 100; and > 500. For data analysis, these seven categories were summarized into four categories (Fig. 1). In the results section, we will especially refer to those tasks performed at least one and more than ten times since graduation. Supervision levels were captured on the basis of the levels of supervision established for postgraduate medical education [15]. The study participants indicated the following: I observed the activity but did not perform it; I performed the activity under direct supervision (supervising physician in the room); I performed the activity autonomously under indirect supervision (supervising physician on the ward, readily available); and I performed the activity autonomously under distant supervision (supervising physician not in the hospital, not readily available).

We expected the large majority of beginning residents to have performed the activities within the first months of residency at least under level 3, or indirect supervision [5]. As our study is explorative in nature, we decided to take > 75% as the threshold for operationalizing the large majority. In addition, we report the study results for the plain majority (> 50%).

### Statistical analysis

Analyses were carried out using SPSS 25 [16]. The frequency distribution of the EPA variables is displayed by means of valid percentages, excluding the randomly occurring missing values. Spearman’s Rho correlations were used to determine the relation among the EPA variables. Due to the explorative nature of the analyses, no corrections for multiple testing were applied [17].

## Results

### Participants

In total, 215 graduates returned the questionnaire (return rate = 30%). Of those, 84 were not yet working as physicians or reported insufficiently on the EPA section of the survey. Accordingly, data from 131 graduates were included in this study (response rate = 18%). The graduates were on average 29 years old (SD = 4), and 63% were female. The majority worked in hospitals (50%) or in university medical centres (30%). They were situated in various medical disciplines: internal medicine (*n* = 23), paediatrics (*n* = 15), gynaecology and obstetrics (*n* = 14), anaesthesiology (*n* = 14), general medicine (*n* = 12), surgery (*n* = 12), neurology (*n* = 8), radiology (*n* = 8), psychiatry (*n* = 6), and dermatology (*n* = 5).

### Frequency of performing the EPAs

Figure 1 displays how frequently the participants performed the EPAs since the start of residency. The results are separately depicted for the Core EPAs, the Core medical procedures and the more advanced EPAs.

Among the Core EPAs, 10 out of 11 had been performed at least once by more than 75% of the graduates since the start of residency. The Core EPA “Case presentation” had been carried out by approximately two-thirds of the graduates. Eight out of 11 EPAs had been performed more than 10 times by at least 75% of the graduates. The Core EPAs “Act in emergency situations” and “Case presentation” had been carried out by approximately one-third of the graduates more than 10 times.

Regarding the Core medical procedures, a greater variation was seen in the frequency with which they had been performed by the entering residents. Three out of 13 Core medical procedures had been performed at least once by more than 75% of the graduates since the start of residency. Eight procedures had been performed by at least 50% of the graduates. Seven of the 13 Core medical procedures had been performed by less than 25% of the entering graduates more than 10 times. The lowest performance rate (34%) was seen for the Core medical procedure “Nasogastric tube”. Ten procedures had not been performed by at least one-third of the participants.

Among the more advanced EPAs, none of the 5 professional activities had been carried out at least once by more than 75% of the graduates. All the tasks had been carried out by at least 50% of the graduates, three more than 10 times.

### Experienced supervision level

**Fig. 2:**
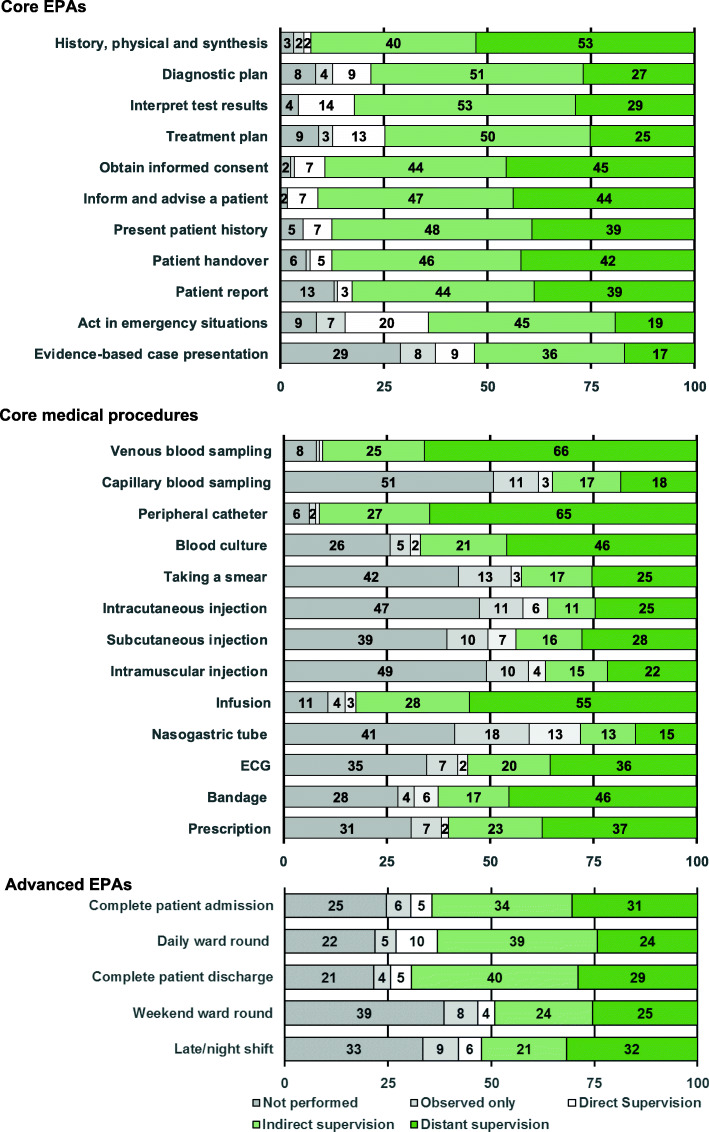
Experienced supervision level. The figure depicts the valid percentage of participants performing the EPAs under the respective supervision levels

Figure 2 depicts the supervision levels under which participants performed the EPAs, including the percentage of participants who had not performed the activities and had only observed them.

Among the Core EPAs, 9 of the 11 had been performed by more than 75% of respondents, i.e., the large majority of graduates, under the expected level of supervision. The exceptions were again the EPAs “Act in emergency situations” and “Evidence-based case presentation”, which had been carried out by 64 and 53% of participants under indirect or distant supervision, respectively.

Regarding the Core medical procedures, greater variability in workplace practice was again observed. Three out of the 13 Core medical procedures had been performed under at least indirect supervision by more than 75% of the graduates since the start of the residency and 5 procedures by at least 50% of the graduates.

Regarding the more advanced EPAs, none of the tasks had been carried out by more than 75% of the graduates under at least indirect supervision. For 4 out of 5, this supervision level was reached by at least 50% of the graduates.

### Exploratory analyses

The statistical analyses revealed that the history of performing the activities correlated positively with the experienced supervision level for all activities. The correlation coefficients ranged between 0.211 and 0.77 (Additional file 2).

## Discussion

The purpose of this study was to gather empirical information on beginning residents’ actual task performance and supervision level using a regular post-graduation survey. In the following, we will exemplarily triangulate the results of this survey to the expert expectations, both from the same institution, thereby combining several methods to reflect on the content of our Core EPAs from an empirical angle.

It was among the Core EPAs that we found the best fit between the expectations of the medical experts and the actual workplace performance of the beginning residents. For nine of the 11 Core EPAs, the tasks were performed by more than 75% of the graduates under at least indirect supervision. This supports the content validity of these tasks and their granularity and further indicates that these tasks are relevant to many if not all covered medical disciplines. The Core EPAs “Act in emergency situations” and “Evidence-based case presentation” were performed more infrequently and under a closer level of supervision by the majority of graduates. However, there are still valid reasons to include both activities in the set of Core EPAs for our institution. In the clinical workplace, graduating physicians can at any time be exposed to patients who suddenly develop life-threating conditions at a time when no supervisor is in the room or on the ward. Graduates must be able to recognize these situations and provide appropriate patient care until the emergency team or the supervisor has arrived. The Core EPA “Evidence-based case presentation” might not be as essential as the EPA “Act in emergency situations”. However, in our context, all the medical experts contributing to the EPA content definition were working in an academic setting. It was important to them to include at least one EPA that explicitly applies science, i.e., evidence-based medicine, to patient care, both to reflect the practice in their clinical context and to impact the future clinical practice of Charité graduates in other settings [5, 14].

Among the Core medical procedures, we found only a marginal fit between the medical experts’ expectations and the actual practice of graduating physicians in our workplace context. Three of the 13 Core medical procedures had been carried out by more than 75% of the graduates under at least indirect supervision. Furthermore, 10 of the 13 procedures had not been carried out by more than one-third of the graduates. This may reflect the fact that most of the tasks included are rarely performed at all and may be performed by other professionals and with variable frequency in the various disciplines. This raises a question regarding whether all the identified procedures qualify as Core medical procedures expected of all graduates at our institution. Internationally, the number of Core medical procedures varies between 6 and 22; thus, there are evidently some differences in the number and kind of medical procedures considered to be important [1, 3]. The empirical results of this study serve as an important stimulus for our institution to consider a substantial reduction in the number of Core medical procedures expected to be performed by its graduates under indirect supervision. This does not mean these tasks should not be further trained and assessed during undergraduate medical training. In many cases, it could lead to the adjustment of the supervision level being expected for entry into residency. In disciplines and contexts in which the procedures are not part of general practice, co-performance or direct supervision may be a more reasonable starting point. The present study did not allow to compare how often and under what level of supervision the procedures are conducted in the different disciplines due to the rather low overall sample size. To address these issues in future studies, we have now permanently included the EPA section in the post-graduation survey and will use the accumulating data for such comparative analysis. In combination with information from different sources and studies, these results will be used to discuss and decide on the refinement of Core EPA for entry into residency in our context.

Among the advanced EPAs, 4 out of 5 had been performed by more than 50% of the graduates. For the interpretation of these results, several factors should be considered. The more advanced EPAs included in this study were not defined as outcomes for the undergraduate medical curricula by the medical experts but represent broader activities integrating and embracing, in a nested manner, several smaller EPAs [7]. As the responsibility of entering residents increases quickly in the first months of their postgraduate training, it is not surprising that depending on context, ability and discipline, a relevant number of graduates are already carrying out more advanced EPAs a few months after the start of residency. However, based on our empirical study, we would not suggest that the frequency of performance of the advanced EPAs qualifies them to be considered Core EPAs for entering into residency. It does not seem realistic to expect that the majority of entering residents can be fully entrusted to carry out, for instance, a complete patient admission. If so, this would mean, for example, that without confirmation from a supervisor, the entering graduate, based on his/her interpretation of the patient’s history and physical examination, could autonomously decide on far-reaching diagnostic or therapeutic steps, such as ordering a CT scan with contrast media or sending a patient to the surgery theatre. However, the results of this study may indicate that the definition of Core EPAs for entering into residency, despite all its advantages, may be too narrow. While Core EPAs help students to master the transition from undergraduate to postgraduate medical training, there is a need to specify how the Core EPAs for entry into residency feed into the larger, more advanced EPAs residents have to carry out a few months later. This is in line with the recommendations by Chen and colleagues, who emphasized the continuity of medical education and pleaded for an overarching curriculum covering both under- and postgraduate education [18].

Research focusing on the perceived preparedness of beginning residents to perform Core EPAs, from the perspectives of both residents and supervising physicians, discusses the need to revise undergraduate curricula to better prepare students for medical practice [10–13, 19]. We can support those recommendations. Unsurprisingly, our study found that the frequency of performing an activity is positively associated with the level of entrustment. The integration of the Core EPAs and procedures into undergraduate curricula requires the opportunity to practice the activities in order to ease the critical transition to postgraduate education for graduating physicians [20, 21]. Another notable result of this study is that a regular post-graduation survey provides tangible, sufficiently meaningful information to enhance and reflect on the content validity of the Core EPAs for a given institution and context.

This study has several limitations. First, the study was conducted at a single medical school. The transferability of our findings to other contexts needs to be shown in future studies. Second, the questions covering the EPAs were not pilot tested with graduates, which could have led to a valuable refinement of the questionnaire. Third, the response rate in this voluntary post-graduation survey was 18%. While this is not unusual for medical education research [22], it may have created selection bias. Despite this, we feel that the results obtained provided tangible and sufficiently meaningful information to reflect on and enhance the content validity of our institutional Core EPAs for entry into residency. Finally, we did not apply the full entrustment scale as proposed in the literature [15]. We adjusted the scale to our expected level of supervision for graduates, but the administration of the complete scale might have resulted in different findings.

## Conclusion

The presented study provides empirical information on the workplace participation of entering residents by means of a set of institutional Core EPAs and procedures. It adds complementary evidence on the validity of the professional activities our medical graduates should be trained in during undergraduate education. The results of this study and the chosen methodological approach may guide and assist medical faculties and organizations in enhancing the content validity of Core EPAs for entry into residency.

## Supplementary Information

**Additional file 1.** Questionnaire extract: Questions regarding the Charité EPAs

**Additional file 2.** Correlations between the EPA variables for Core EPAs, Core procedures and advanced EPAs

## Data Availability

The dataset used and analysed during this study is available from the corresponding author on request where warranted.

## Abbreviations

EPAs: Entrustable professional activities
