# Supplementary material for: What can we expect from medical graduates? Empirical survey on the performance of Core EPAs in the first days of residency
Source: BMC Med Educ. 2020 Nov 23;20:452. doi: 10.1186/s12909-020-02376-y (PMC7685603; doi:10.1186/s12909-020-02376-y)
Supplement: Supplementary file 1 — Additional file 1. Questionnaire extract: Questions regarding the Charité EPAs [file 12909_2020_2376_MOESM1_ESM.docx]

**Questionnaire extract: Questions regarding the Charité EPAs**

How many times did you perform the following activities since the start of your residency?

|  | **Not once** | **1-5 times** | **6-10 times** | **11-25 times** | **26-100 times** | **>100 times** | **>500 times** | **No information** |
| --- | --- | --- | --- | --- | --- | --- | --- | --- |
| **Along the clinical encounter** |  |  |  |  |  |  |  |  |
| Take a medical history, perform a physical examination and summarize the results in a structured manner (typical presentation, common disease pattern) |  |  |  |  |  |  |  |  |
| Compile a diagnostic plan and initiate implementation (typical presentation, common disease pattern, typical course of disease; tiered diagnostics) |  |  |  |  |  |  |  |  |
| Interpret test results and initiate further steps (common diagnostic methods) |  |  |  |  |  |  |  |  |
| Compile a treatment plan and initiate implementation (common disease pattern, typical course of disease) |  |  |  |  |  |  |  |  |
| **General medical procedures** |  |  |  |  |  |  |  |  |
| Venous blood sampling |  |  |  |  |  |  |  |  |
| Capillary blood sampling |  |  |  |  |  |  |  |  |
| Inserting a peripheral catheter |  |  |  |  |  |  |  |  |
| Taking a blood culture |  |  |  |  |  |  |  |  |
| Taking a smear (oral, nasal, wound, anal, urogenital) |  |  |  |  |  |  |  |  |
| Giving an intracutaneous injection |  |  |  |  |  |  |  |  |
| Giving a subcutaneous injection |  |  |  |  |  |  |  |  |
| Giving an intramuscular injection |  |  |  |  |  |  |  |  |
| Giving an infusion |  |  |  |  |  |  |  |  |
| Placing a nasogastric tube |  |  |  |  |  |  |  |  |
| Taking an ECG |  |  |  |  |  |  |  |  |
| Putting on or changing a bandage |  |  |  |  |  |  |  |  |
| Writing a prescription |  |  |  |  |  |  |  |  |
| **Communication with patients** |  |  |  |  |  |  |  |  |
| Seek consent for medical procedures and diagnostics (inform patient about course, benefits, risks and alternatives) |  |  |  |  |  |  |  |  |
| Inform and advise patients (common consolations, reasons and diseases) |  |  |  |  |  |  |  |  |
| **Communication and collaboration with colleagues** |  |  |  |  |  |  |  |  |
| Present a patient history (structured; according to the target audience and situational requirements) |  |  |  |  |  |  |  |  |
| Give or receive a patient handover (structured; according to the target audience and situational requirements) |  |  |  |  |  |  |  |  |
| Write and transmit a patient report (structured; transmit oneself or delegate) |  |  |  |  |  |  |  |  |
| **Additional professional activities** |  |  |  |  |  |  |  |  |
| Recognize an emergency situation and act upon it (estimate the degree of severity, provide on-the-spot aid, call for help) |  |  |  |  |  |  |  |  |
| Undertake an evidence-based patient case and initiate patient-specific implementation |  |  |  |  |  |  |  |  |
| Manage an in-patient admission |  |  |  |  |  |  |  |  |
| Conduct a ward round in the hospital |  |  |  |  |  |  |  |  |
| Manage an in-patient discharge |  |  |  |  |  |  |  |  |
| Conduct a weekend ward round in the hospital |  |  |  |  |  |  |  |  |
| Take a late/night shift (supervising physician available via telephone) |  |  |  |  |  |  |  |  |

Please indicate the highest level of supervision under which you performed the respective EPA at least three times since the start of your residency.

Level 1: I observed the activity but did not perform it

Level 2: I performed the activity under direct supervision (supervising physician in the room)

Level 3: I performed the activity autonomously under indirect supervision (supervising physician on the ward, readily available)

Level 4: I performed the activity autonomously under distant supervision (supervising physician not in the hospital, not readily available).

|  | **Level 1** | **Level 2** | **Level 3** | **Level 4** | **No information** |
| --- | --- | --- | --- | --- | --- |
| **Along the clinical encounter** |  |  |  |  |  |
| Take a medical history, perform a physical examination and summarize the results in a structured manner (typical presentation, common disease pattern) |  |  |  |  |  |
| Compile a diagnostic plan and initiate implementation (typical presentation, common disease pattern, typical course of disease; tiered diagnostics) |  |  |  |  |  |
| Interpret test results and initiate further steps (common diagnostic methods) |  |  |  |  |  |
| Compile a treatment plan and initiate implementation (common disease pattern, typical course of disease) |  |  |  |  |  |
| **General medical procedures** |  |  |  |  |  |
| Venous blood sampling |  |  |  |  |  |
| Capillary blood sampling |  |  |  |  |  |
| Inserting a peripheral catheter |  |  |  |  |  |
| Taking a blood culture |  |  |  |  |  |
| Taking a smear (oral, nasal, wound, anal, urogenital) |  |  |  |  |  |
| Giving an intracutaneous injection |  |  |  |  |  |
| Giving a subcutaneous injection |  |  |  |  |  |
| Giving an intramuscular injection |  |  |  |  |  |
| Giving an infusion |  |  |  |  |  |
| Placing a nasogastric tube |  |  |  |  |  |
| Taking an ECG |  |  |  |  |  |
| Putting on or changing a bandage |  |  |  |  |  |
| Writing a prescription |  |  |  |  |  |
| **Communication with patients** |  |  |  |  |  |
| Seek consent for medical procedures and diagnostics (inform patient about course, benefits, risks and alternatives) |  |  |  |  |  |
| Inform and advise patients (common consolations, reasons and diseases) |  |  |  |  |  |
| **Communication and collaboration with colleagues** |  |  |  |  |  |
| Present a patient history (structured; according to the target audience and situational requirements) |  |  |  |  |  |
| Give or receive a patient handover (structured; according to the target audience and situational requirements) |  |  |  |  |  |
| Write and transmit a patient report (structured; transmit oneself or delegate) |  |  |  |  |  |
| **Additional professional activities** |  |  |  |  |  |
| Recognize an emergency situation and act upon it (estimate the degree of severity, provide on-the-spot aid, call for help) |  |  |  |  |  |
| Undertake an evidence-based patient case and initiate patient-specific implementation |  |  |  |  |  |
| Manage an in-patient admission |  |  |  |  |  |
| Conduct a ward round in the hospital |  |  |  |  |  |
| Manage an in-patient discharge |  |  |  |  |  |
| Conduct a weekend ward round in the hospital |  |  |  |  |  |
| Take a late/night shift (supervising physician available via telephone) |  |  |  |  |  |
