# Supplementary material for: What can we expect from medical graduates? Empirical survey on the performance of Core EPAs in the first days of residency
Source: BMC Med Educ. 2020 Nov 23;20:452. doi: 10.1186/s12909-020-02376-y (PMC7685603; doi:10.1186/s12909-020-02376-y)
Supplement: Supplementary file 2 — Additional file 2. Correlations between the EPA variables for Core EPAs, Core procedures and advanced EPAs [file 12909_2020_2376_MOESM2_ESM.docx]

Correlations between the EPA variables for Core EPAs, Core procedures and advanced EPAs

|  | **Frequency &**  **Supervision level** |
| --- | --- |
| **Core EPAs** | |
| History, physical and synthesis | .36** |
| Diagnostic plan | .52** |
| Interpret test results | .48** |
| Treatment plan | .46** |
| Obtain informed consent | .48** |
| Inform and advise a patient | .32** |
| Present patient history | .21* |
| Patient handover | .28** |
| Patient report | .25* |
| Act in emergency situations | .50** |
| Evidence-based case presentation | .50** |
| **Core Procedures** | |
| Venous blood sampling | .28** |
| Capillary blood sampling | .49** |
| Peripheral catheter | .32** |
| Blood culture | .55** |
| Taking a smear | .58** |
| Intracutaneous injection | .69** |
| Subcutaneous injection | .62** |
| Intramuscular injection | .52** |
| Infusion | .33** |
| Nasogastric tube | .77** |
| ECG | .42** |
| Bandage | .47** |
| Prescription | .31** |
| **Advanced EPAs** | |
| Complete patient admission | .61** |
| Ward round | .47** |
| Weekend ward round | .55** |
| Complete patient discharge | .63** |
| Late/night shift | .54** |

Spearman’s Rho Correlation: **p < 0.01. *p < 0.05

1) Frequency of performing EPAs; 2) Supervision level when performing EPAs
